# Phosphorescence Monitoring of Hypoxic Microenvironment in Solid-Tumors to Evaluate Chemotherapeutic Effects Using the Hypoxia-Sensitive Iridium (III) Coordination Compound

**DOI:** 10.1371/journal.pone.0121293

**Published:** 2015-03-18

**Authors:** Yun Zeng, Yang Liu, Jin Shang, Jingwen Ma, Rong Wang, Lei Deng, Youmin Guo, Fan Zhong, Mingfeng Bai, Shaojuan Zhang, Daocheng Wu

**Affiliations:** 1 The Key Laboratory of Biomedical Information Engineering, Ministry of Education, School of Life Science and Technology, Xi’an Jiaotong University, Xi’an, P. R. China; 2 School of Life Sciences and Institutes of Biomedical Sciences, Fudan University, Shanghai, P. R. China; 3 Radiology Department, First Affiliated Hospital, Xi’an Jiaotong University, Xi’an, P. R. China; 4 Molecular Imaging Laboratory, Department of Radiology, School of Medicine, University of Pittsburgh, Pittsburgh, Pennsylvania, United States of America; Osaka University, JAPAN

## Abstract

**Objectives:**

To utilize phosphorescence to monitor hypoxic microenvironment in solid-tumors and investigate cancer chemotherapeutic effects *in vivo*.

**Methods:**

A hypoxia-sensitive probe named BTP was used to monitor hypoxic microenvironment in solid-tumors. The low-dose metronomic treatment with cisplatin was used in anti-angiogenetic chemotherapeutic programs. The phosphorescence properties of BTP were detected by a spectrofluorometer. BTP cytotoxicity utilized cell necrosis and apoptosis, which were evaluated by trypan blue dye exclusion and Hoechst33342 plus propidium iodide assays. Tumor-bearing mouse models of colon adenocarcinoma were used for tumor imaging *in vivo*. Monitoring of the hypoxic microenvironment in tumors was performed with a Maestro 2 fluorescence imaging system. Tumor tissues in each group were harvested regularly and treated with pathological hematoxylin and eosin and immunohistochemical staining to confirm imaging results.

**Results:**

BTP did not feature obvious cytotoxicity for cells, and tumor growth in low-dose metronomic cisplatin treated mice was significantly inhibited by chemotherapy. Hypoxic levels significantly increased due to cisplatin, as proven by the expression level of related proteins. Phosphorescence intensity in the tumors of mice in the cisplatin group was stronger and showed higher contrast than that in tumors of saline treated mice.

**Conclusions**

We develop a useful phosphorescence method to evaluate the chemotherapeutic effects of cisplatin. The proposed method shows potential as a phosphorescence imaging approach for evaluating chemotherapeutic effects *in vivo*, especially anti-angiogenesis.

## Introduction

Tumor is an extremely common problem for human beings, and chemotherapy is still the main treatment measure for tumor therapy. In clinical trials, the tumor size is a main and superficial characteristic without monitoring of microenvironment. As investigators do not know more if they don’t have complicated analysis, they can neither evaluate chemotherapeutic effects in perspective nor design new chemotherapy regimens to predict its effects. Therefore, a reasonable method is urgently needed for evaluating chemotherapeutic effects. To develop such a method, a specific landmark of tumor which is significantly different with normal tissues should be indicated.

Recent studies found hypoxia is a key feature in the solid-tumor microenvironment [1], facilitates tumor progression, metastatic spread, and resistance to radiation and chemotherapy. Hypoxia-inducible factor 1 (HIF-1) is the most significant hypoxia-related protein, and its cellular signal pathway has been studied for many years [2]. HIF-1 can activate transcription of many genes involved in angiogenesis, cell growth and survival, glucose metabolism, invasion, metastasis, and drug resistance, to name a few [3]. Angiogenesis-related protein vascular endothelial growth factor (VEGF) and multidrug resistance 1 (MDR1) are up-regulated by hypoxic conditions in tumor cells. Angiogenesis is mostly an adaptive response to tissue hypoxia, and also an essential requirement for neoplastic growth [4]. Hence, hypoxia and angiogenesis are hallmarks of cancer and major targets in cancer therapy, and several target-hypoxia/angiogenesis agents are being actively studied for their antitumor activity in preclinical models and/or in clinical trials [5].

The impact of chemotherapy on the tumor oxygenation environment remains controversial. Early work from pioneers, such as computed tomography (CT) perfusion [6] and dynamic contrast-enhanced magnetic resonance imaging (MRI) [7], suggest that reduction of newly formed blood vessels starves tumors from nutrients and oxygen, thereby reducing tumor growth and inducing tumor cell death (starvation hypothesis). According to the Jain’s hypothesis, treatment with bevacizumab can primarily improve chemotherapeutic efficacy by normalizing the tumor vasculature, resulting in more effective oxygen and drug delivery [8]. What is clear in these tumor studies is that the mechanisms of chemotherapy are fairly complex and depend on the tumor type, hypoxia detection time point, and hypoxia modality [9]. Various cytotoxic chemotherapeutic agents have anti-angiogenesis effects [10, 11], and low-dose metronomic chemotherapeutic treatment with cisplatin can affect angiogenesis [12, 13]. To date, however, intuitive and direct methods for detecting changes in tumor hypoxic microenvironment resulting from chemotherapy are limited [14–16].

Several measures have been developed to observe and measure hypoxic microenvironment. The most accurate method involves the use of an oxygen electrode to penetrate the tumor tissue [17]; this method, however, is invasive and may only measure O_2_ in tissues where penetration occurs. As well, the oxygen electrode may directly consume O_2_. Therefore, noninvasive approaches are necessary to monitor hypoxia in tumors both accurately and reliably.

A number of noninvasive imaging techniques for measuring tumor hypoxia have been exploited, including radionuclide imaging (positron emission tomography [PET] and single-photon emission computed tomography [SPECT]), MRI, and optical imaging [18]. In hypoxia monitoring, nitroimidazole compounds, such as fluoromisonidazole (FMISO) [19] and fluoroazomycinarabinoside (FAZA) [20], have been developed as hypoxia-targeting PET probes for noninvasive hypoxia monitoring. Cu (II)-ATSM is another PET probe without nitroimidazole. These probes radiolabeled with radioisotope can be reduced by enzyme systems in hypoxic tissue, which is believed to bind to macromolecules [21–23]. Among these imaging techniques, optical imaging has several advantages, including high sensitivity and resolution, low imaging cost, and lack of radioisotope requirement. Phosphorescent coordination compounds show luminescent emissions that are distinct from those of the tumor hypoxia targeting probes described above. They are distributed systemically through the whole body and emit phosphorescence where the oxygen supply is insufficient.

Iridium (III) coordination compounds are superior to other phosphorescence-emitting probes, such as platinum (II)- and palladium (II)-porphyrins, and ruthenium (II) coordination compounds, because of their small size and suitable phosphorescence lifetime of the former; these characteristics are favorable for oxygen sensing in living animals [24]. Furthermore, the optical properties of iridium (III) coordination compounds may be tuned by adjusting ancillary ligands [25].

An effective red phosphorescence-emitting probe BTP, [bis(2-(2’-benzothienyl)pyridinato-N, C^3^’) iridium (acetylacetonate)] [25] was used in the medical field [26] and developed using nanotechnology [27] in our earlier work. It is quiescent in normoxic tissues but become emissive in hypoxic regions because of their oxygen-dependent triplet state (oxygen quenching). This finding provides opportunities for hypoxia imaging with high contrast [26].

**Fig 1:**
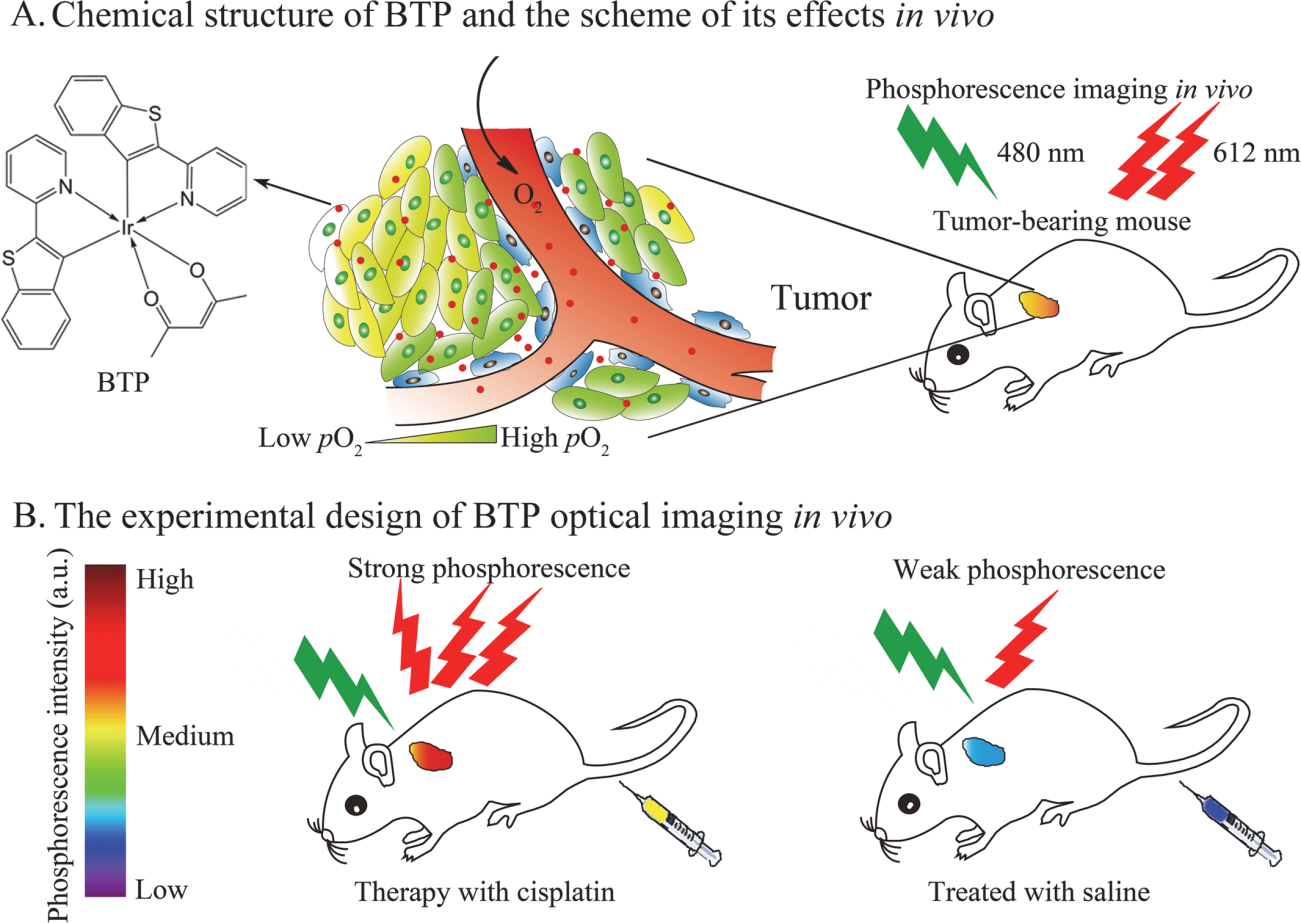
The schematic diagram of our research. (A) Chemical structure of BTP and the scheme of its effects *in vivo*. (B) The experimental design of BTP optical imaging for monitoring tumor hypoxic microenvironment *in vivo* in the cisplatin therapy process.

The purpose of the present study is to develop a phosphorescence imaging approach using BTP to monitor hypoxic changes resulting from chemotherapeutic treatment *in vivo*. We hypothesize that the hypoxia-sensitive probe BTP is useful for quantitatively evaluating changes in tumor hypoxia during chemotherapy. We designed an experiment using BTP phosphorescence imaging to monitor tumor hypoxic microenvironment *in vivo* during cisplatin treatment to assess chemotherapeutic effects and the reasonability of treatment (Fig. 1). The results of this paper agreed with our hypothesis; this sensor showed great potential for hypoxia-targeting cancer imaging *in vitro* and *in vivo*, and might be used as a potent biomarker for real-time monitoring of hypoxic microenvironment and detection of overall changes resulting from chemotherapeutic treatment. Therefore, BTP may be applied as a promising and novel phosphorescence imaging method for evaluating chemotherapeutic effects *in vivo*.

## Materials and Methods

### Materials

BTP synthesized according to the literature was purchased from American Dye Source, Inc. Mouse monoclonal antibodies to HIF-1α and VEGF were purchased from Sigma-Aldrich Trading Co., Ltd. (Shanghai, China). Other reagents were purchased from Aladdin Chemical Reagents Co., Ltd. All reagents were of analytical grade and directly used without further purification.

A mouse colon adenocarcinoma (CT-26) cell line [28] was purchased from the Chinese Academy of Sciences (Shanghai). Cell culture consumables were purchased from Thermo Fisher Scientific Co., Ltd. (Shanghai, China). A streptavidin-biotin2 system labeled with horseradish peroxidase (LSAB2 System-HRP) was purchased from Dako Co., Ltd. Male nude BALB/c mice were purchased from the Department of Experimental Animals, Shanghai Medical College, Fudan University. The mice were raised under a standard housing environment until 6 weeks of age and featured body weights of at least 25.0 g. All animal experiments were approved by the Ethics Committees of Xi'an Jiaotong University.

### Phosphorescence properties of BTP

A spectrofluorometer (FluoroMax-4, HORIBA JobinYvon, France) was used to measure the phosphorescence spectra of BTP under different conditions (hypoxia and normoxia). Briefly, 5 mg of BTP was dissolved in 35 mL of DMSO; 3 mL of this mixture was pipetted into a quartz cuvette. Phosphorescence excitation spectroscopy was performed at an excitation wavelength of 480 nm.

To observe the phosphorescent performance of BTP in a hypoxic atmosphere, two quartz cuvettes with 3.0 mL of BTP were placed under a handheld UV lamp at an excitation wavelength of 365 nm in a dark room. Photographs of the cuvettes were obtained by a camera (Powershot A650IS, Canon, Japan). Nitrogen was used to purge the air out from one of the quartz cuvettes; the other tube was used as a control. In both tubes, the BTP concentration was set to 0.14 mg·mL^−1^.

### Cytotoxicity evaluation of BTP

To evaluate the cytotoxicity of BTP, CT-26 cell necrosis and apoptosis were evaluated in cell cultures to which BTP had been added for up to 72 h using trypan blue dye exclusion and Hoechst 33342 plus propidium iodide (PI) assays. BTP concentrations of 0, 1, 5, 20, 50, 100 μM were tested. Cholesterol was used as a negative control and 7-ketocholesterol [29] was used as a positive control; both molecules feature lipophilicity similar to that of BTP [30].

### Cell uptake of BTP

CT-26 cells were cultured in DMEM medium containing 25 mmol·L^−1^ of glucose with 10% FBS at 37°C and then cultured under 5% or 20% O_2_ in a concentration-changeable multi-gas incubator (MCO-5M, Sanyo, Japan) for 24 h at 37°C. Afterward, BTP dissolved in DMSO was added to the medium at a final concentration of 5 μM for 2 h. An inverted fluorescence microscope (Eclipse Ti-S, Nikon, Japan) with a camera was used to obtain phosphorescence images.

### Nude BALB/c mice model of colon adenocarcinoma

Tumor transplants were established in male nude BALB/c mice by injection of 5×10^6^ mouse colon adenocarcinoma-derived CT-26 cells to the left anterior axilla. Experiments with tumor-bearing mice were performed one week after injection of tumor cells.

### Low-dose metronomic chemotherapy treatment with cisplatin

To evaluate hypoxic changes resulting from chemotherapy, all mice were treated for 21 days with low-dose metronomic cisplatin, a well-studied anti-cancer chemotherapeutic drug commonly used against different human tumors. When the tumor volumes approached 60–70 mm^3^, saline (100 μL) and cisplatin (1 mg·kg^−1^, 100 μL) were intraperitoneally injected into the 12 CT-26 tumor-bearing nude mice every two days (6 with saline, 6 with cisplatin).

### Monitoring tumor hypoxic microenvironment *in vivo*

The tumor size in each mouse was measured every three days after tumor implantation by a caliper and calculated as follows: volume = (tumor length) × (tumor width)^2^/2. Every three days, hypoxic changes in tumors *in vivo* were monitored by carrying out optical imaging, beginning from day nine post-treatment as described above. BTP DMSO/saline solution was injected intravenously into the animals 2 h before imaging. Phosphorescent images of the whole mouse body as well as a close-up image were obtained using an *in vivo* fluorescence imaging system (Maestro 2, CRI, USA) under the following conditions: “Blue” filter model; excitation filter range, 455 nm (435–480 nm); emission filter range, 490 nm longpass; acquisition setting, 500 to 720 nm in 10 nm steps. The auto-fluorescence of mice and BTP phosphorescence were obtained and then unmixed using Maestro spectral software.

### Pathological and immunohistochemical studies

Tumors were quickly removed and fixed with 4% paraformaldehyde in 0.1 M phosphate buffer. After fixation, tumors were immersed in a series of sucrose solutions with increasing concentrations for 48 h at 4°C. For the pathological study, tumor samples were embedded in Tissue-Tek O. C. T. compound mounting medium (Sakura Finetek Inc., CA, USA), frozen in liquid nitrogen, and cryostat-sectioned (5 μm) by a cryostat (Jung CM3000, Leica, Germany). Histological sections from cryostat blocks were stained with hematoxylin and eosin (H&E). Tissue antigens were retrieved after formalin-fixing and paraffin-embedding, and antigens were stained with the primary antibodies (mouse monoclonal antibodies to HIF-1α or VEGF; 1:200 dilution). A second immune reaction was performed using LSAB2 System-HRP. The quantitative data of IOD SUM were extracted using Image-Pro Plus 6 (Media Cybernetics Co., Ltd., USA).

### Statistical analysis

Data were presented as mean ± SE. Three repetitions or sets were performed. *T*-test was performed to determine differences among samples. A *p* value of less than 0.05 was considered significant (*p<0.05, **p<0.01, ***p<0.001). The Software packages of GraphPad Prism 5 (GraphPad Software, Inc., USA) and SPSS 19.0 (IBM Co., USA) were used in the statistical analysis.

## Results

### Phosphorescence properties of BTP

**Fig 2:**
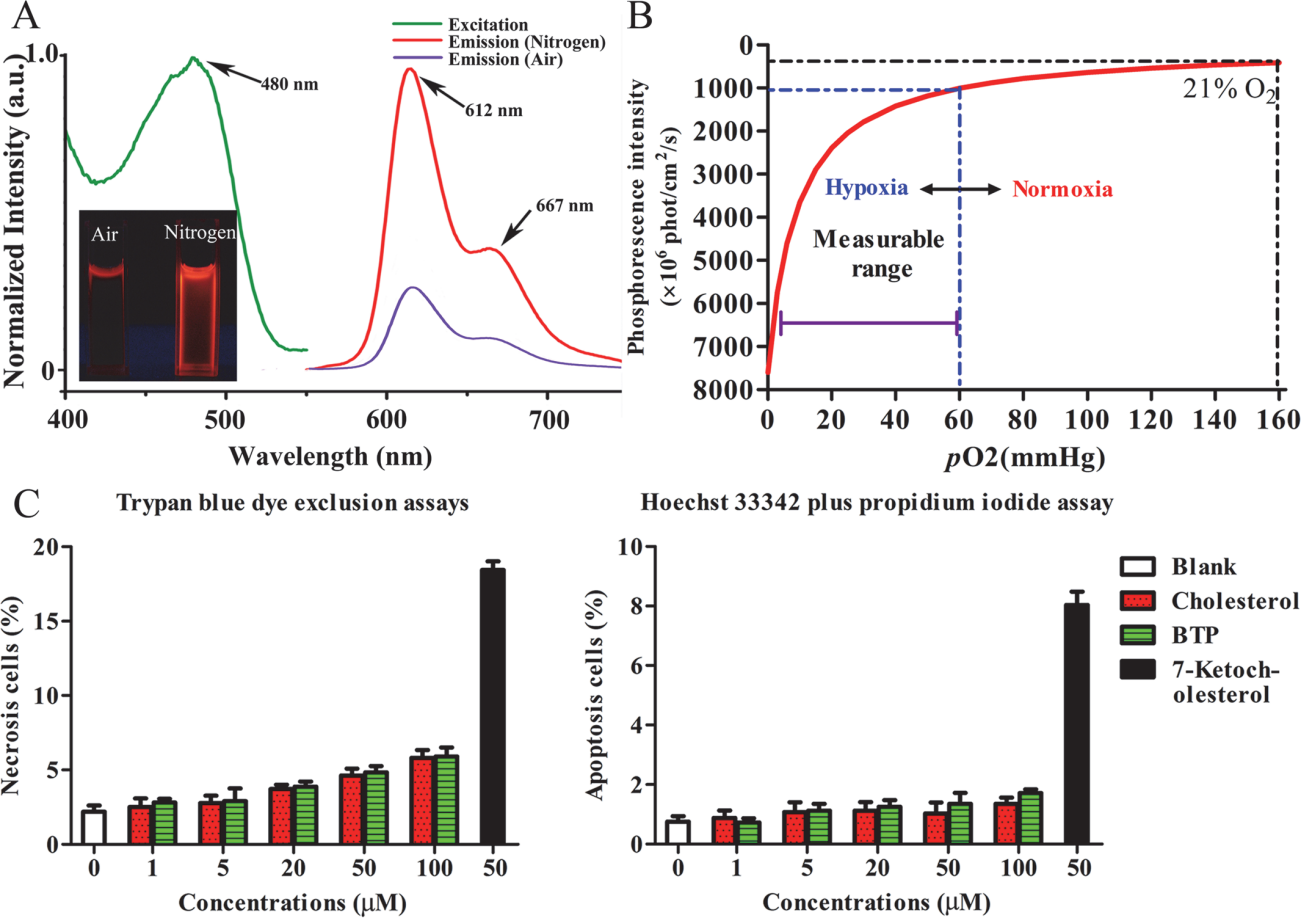
The physical and cytotoxicity properties of BTP. (A) The excitation and emission spectra of BTP; the emission spectra include two curves which are at the air and nitrogen atmospheres, corresponding to two quartz cuvettes, respectively. (B) The relationship between the *p*O2 and phosphoresce intensity. (C) Cytotoxicity evaluation of BTP: Trypan blue dye exclusion assay to measure the cell necrosis rate and Hoechst33342 plus PI assay to measure the cell apoptosis rate (n = 10).

The chemical structure of BTP is shown in Fig. 1. BTP had a broad excitation wavelength ranging from 400 to 550 nm. When excited at its maximum excitation wavelength (480 nm), BTP showed a maximum emission peak at 612 nm, with a shoulder at 667 nm (Fig. 2A). DMSO made the spectra shift red compared with low polarity solvent. Part of the emission curve extends to the near-infrared region (650–900 nm), which is the preferred optical window for *in vivo* imaging because of the relatively low tissue absorption and auto-fluorescence in this region. BTP emits strong phosphorescence where the O_2_ supply is insufficient. The intensity of emission peaks changed under different conditions (hypoxia and normoxia), and the peak intensity of the compound in pure nitrogen was about 3.5 times that in the atmosphere [27].

Phosphorescent images of BTP samples under normoxic (left) and hypoxic (right) conditions are shown in Fig. 2A. After pumping nitrogen through the BTP solution to generate a hypoxic environment, the phosphorescence signals increased significantly in the test quartz cuvettes. The relationship between *p*O2 and phosphoresce intensity are shown in Fig. 2B. According to Stern-Volmer equation, *Q*_0_/*Q* and τ_0_/τ values are proportional to *p*O2, and our results are similar to the report [31]. Therefore, phosphoresce intensity (*Q*) is inversely proportional to *p*O2.

### Cytotoxicity evaluation of BTP

To evaluate the cytotoxicity of BTP, we examined cell viability according to necrosis and apoptosis in CT-26 cells using trypan blue dye-exclusion and Hoechst 33342 plus PI assays. Necrosis and apoptosis of cells treated with BTP were comparable with those observed in cells treated with the cholesterol control (Fig. 2C). Treatment with BTP concentrations of less than 100 μM yielded a cell necrosis rate of less than 10% and a cell apoptosis rate below 2%. In our previous discussion, we postulated that any cytotoxicity observed might be attributed to the presence of organic solvents (DMSO as the solvents of BTP) injected along with BTP. The water soluble nanoparticles containing a BTP derivative was low cytotoxicity to avoid used organic solvent. In fact, BTP does not have obvious cytotoxicity if solvent is used less than 2% in cell medium (v/v), and may be used as a safe phosphorescence probe *in vivo*.

### Cell uptake of BTP

**Fig 3:**
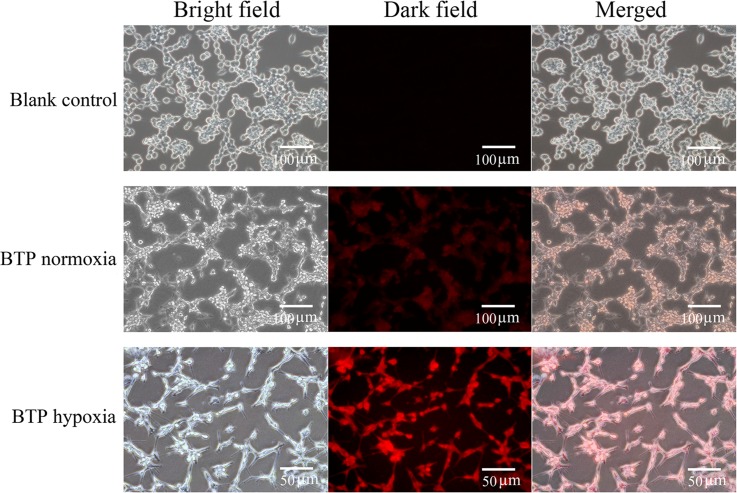
The phosphorescence imaging *in vitro*. CT-26 cells uptake of BTP imaged using an inverted fluorescence microscope as well as a camera, including blank control, BTP under 20% O_2_ (BTP normoxia) and BTP under 5% O_2_ (BTP hypoxia). There are three columns including a bright field, a dark field and a merged field. The first column presents the bright field under an inverted microscope; the second column shows the phosphorescence images of cells as the bright field graphs without any moving, and the third one merged them.

Tumor cell cultures were utilized to examine cell uptake of BTP. CT-26 cells were treated with 5 μM of BTP under 5% (hypoxic) or 20% (normal) O_2_ and cultured for 2 h. Results showed that hypoxic CT-26 cells displayed higher phosphorescence signals than cells under normal *p*O_2_ (Fig. 3).

### Monitoring tumor hypoxic level changes in microenvironment *in vivo* and the pathological and immunohistochemical studies

**Fig 4:**
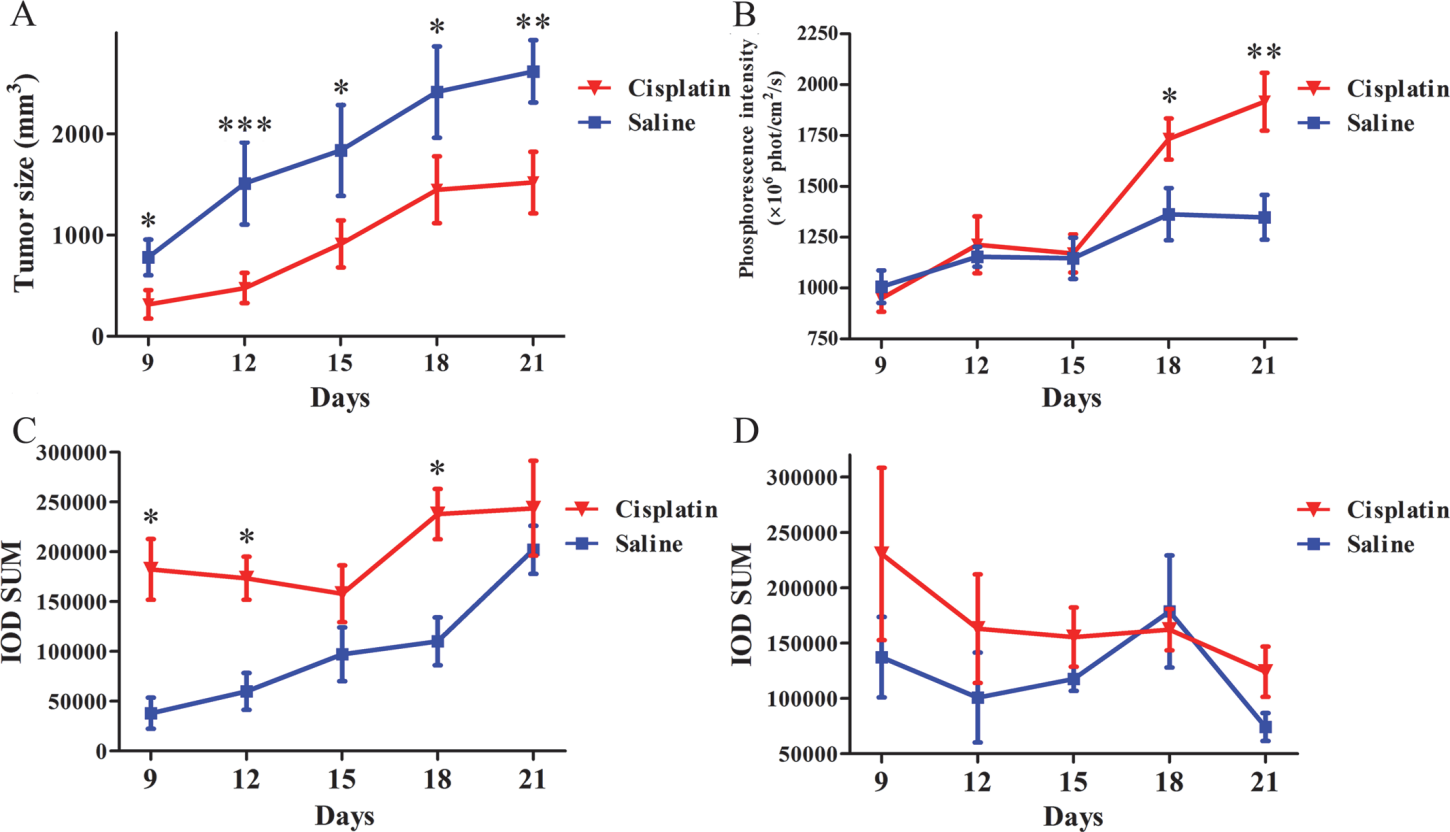
The phosphorescence imaging *in vivo* and immunohistochemical stain. (A) The growth curves of CT-26 xenografts after transplantation into BALB/c nude mice treated with saline and cisplatin (n = 6). (B) The phosphorescence intensity of tumor imaging of CT-26 xenografts after transplantation into BALB/c nude mice treated with saline and cisplatin (n = 6). (C) The quantitative data curves of immunohistochemical stain of HIF-1α in tumor-bearing mice treated with saline and cisplatin, respectively (n = 3). (D) The quantitative data curves of immunohistochemical stain of VEGF in tumor-bearing mice treated with saline and cisplatin, respectively (n = 3). Saline (100 μL) or cisplatin (1 mg·kg^-1^, 100 μL).

**Fig 5:**
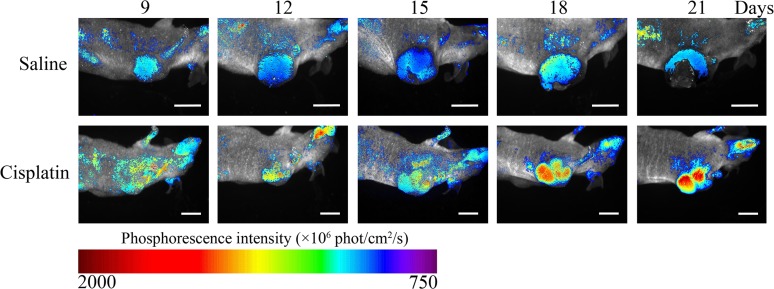
The BTP phosphorescence imaging for monitoring tumor hypoxic microenvironment *in vivo* at different time points (Days 9–21). The cisplatin was used as an anti-tumor agent, and saline was injected as a contrast (n = 6). Scale bar = 10 mm.

**Fig 6:**
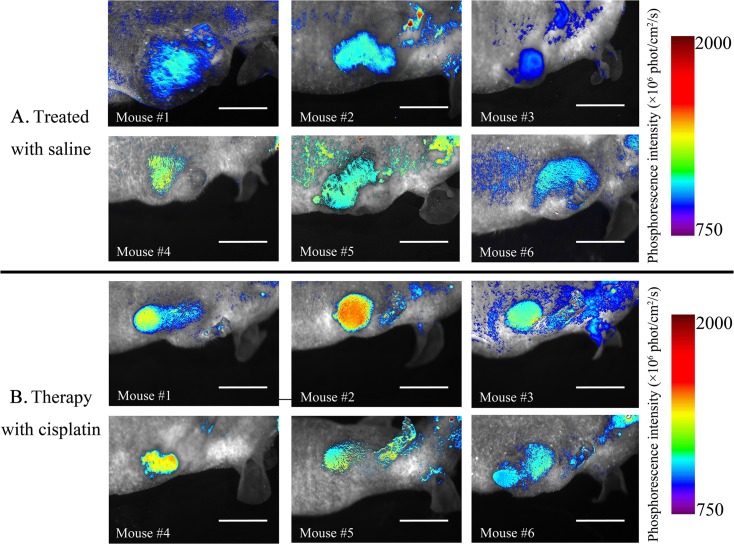
The BTP phosphorescence imaging between cisplatin and saline groups for monitoring tumor hypoxic microenvironment *in vivo* on Day 18 (n = 6). Mice #1–6 are labeled in both cisplatin and saline treatment groups. Scale bar = 10 mm.

CT-26 tumor-bearing nude mice were treated with low-dose metronomic curing with cisplatin, and BTP accumulation in the tumor site was observed using a Maestro 2 fluorescence imaging system. Fig. 4A shows significant inhibition of tumor growth in cisplatin-treated mice (*p*<0.01) compared with that in saline control mice; BTP signals significantly increased over the treatment period (Day 18: *p* = 0.049; Day 21: *p*<0.01). This phenomenon indicates that cisplatin inhibited tumor growth and increased tumor hypoxia. Using the same imaging technique *in vivo*, a mouse treated with saline and another mouse treated with cisplatin were observed continually from Days 9 to 21. Fig. 5 illustrates that the mouse in the saline group showed gradual increases in tumor size as the phosphorescence intensity increased. By contrast, the mouse in the cisplatin group displayed a smaller tumor as well as a sharper increase in phosphorescence intensity. Here, we defined phosphorescence intensity using a color bar: long-wavelength colors present strong intensity while short wavelength colors mean weak intensity. Images of the groups on Day 18 (when significant differences were first observed) are shown in Fig. 6. While the tumor size in mice in the saline group was higher than that in mice in the cisplatin group, the images did not show clear boundaries and the morphology of tumors could not be examined completely. Besides the strong phosphorescence intensity of tumors in mice in the cisplatin group, a high-contrast contour might also be observed, which indicated that O_2_ distribution between tumors and normal tissues was quite different, especially under chemotherapeutic conditions, when changes in the tumor microenvironment might occur.

**Fig 7:**
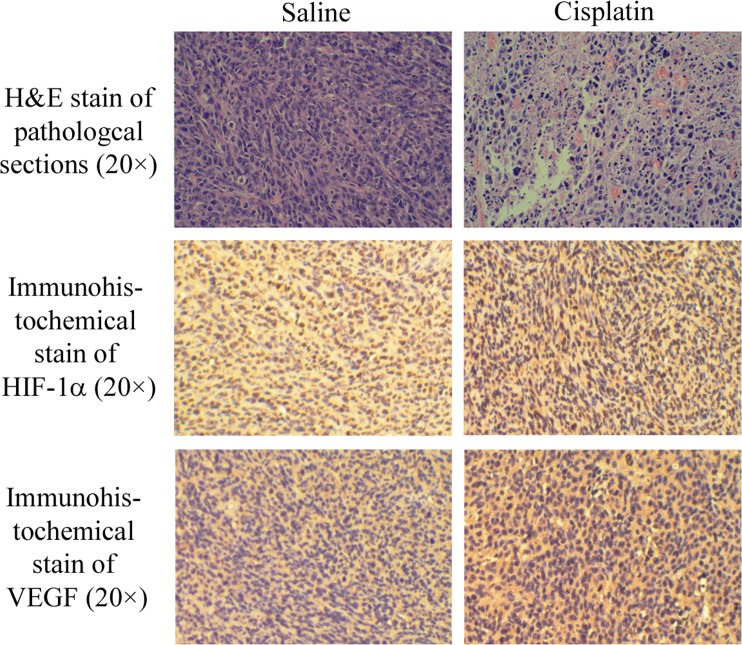
The H&E stain of pathological sections and immunohistochemical stain of mouse HIF-1α and VEGF of tumor tissues on mice treated with saline and cisplatin.

Upon completion of tumor hypoxia imaging, the CT-26 tumor-bearing mice were sacrificed. Tumors were harvested, sectioned, subjected to H&E staining, and then immunohistochemically stained with HIF-1α or VEGF (Fig. 7). Pathological sections illustrate that tumor cells died and tissues appeared empty under chemotherapy. The immunohistochemical stain of HIF-1α shows that HIF-1α expression was higher in the cisplatin group (darker brown area, Day 9: *p* = 0.013; Day 21: *p* = 0.487) than in the saline group, which indicated a positive relationship with hypoxic conditions. The immunohistochemical stain of VEGF shows that VEGF expression was higher in the cisplatin group than in the saline group. However, it had no significant difference between two groups (Day 9: *p* = 0.339; Day 21: *p* = 0.128) for two reasons. One was the suppression of cisplatin, and the other was induced expression by HIF-1. They made VEGF neither increased nor decreased. Quantitative data of IOD SUM were extracted and shown in Fig. 4C and 4D. HIF-1α expressed a trend similar to that of phosphorescence intensity, which showed a linear relationship with O_2_ concentration (Stern-Volmer equation) [32]. HIF-1α expression was always higher in the cisplatin group than that in the saline group. As an up-regulated protein by hypoxia, VEGF expression gradually decreased in the cisplatin group but initially increased and then decreased in the saline group (Fig. 4C and 4D).

## Discussion

Commonly used measurement for tumor hypoxia includes HIF-1α immunohistochemistry assessment, oxygen electrode probe, PET radiotracers such as ^18^F-FMISO, ^18^F-FAZA, Cu (II)-ATSM. Oxygen electrode probe usually provides a minimum invasive measurement for the local oxygen level and may vary largely by the detecting position. In contrast, PET hypoxia imaging can offer an alternative way with non-invasive, overall and accurate hypoxia imaging. However, it may require the high cost facilities and has radiation exposure. In the current study, we used HIF-1α immunohistochemistry staining to validate our findings of BTP-based imaging. However, it is beyond the scope of the current study. Further study may involve a dual model imaging (BTP and PET hypoxia imaging) to validate the oxygen level in tumor area.

**Fig 8:**
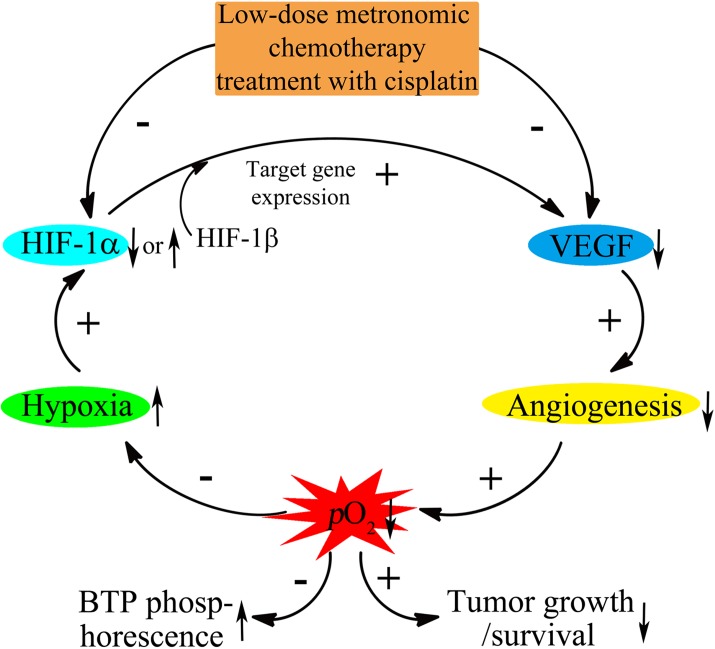
The HIF-1α/VEGF cellular signal pathway and their relationship to *p*O2 and BTP phosphorescence.

To explain the various phenomena in our research, we focus on the HIF-1 cellular signal pathway, which has been investigated for many years [2]. HIF-1 can activate transcription of many genes involved angiogenesis, cell growth and survival, glucose metabolism, invasion, metastasis, and drug resistance, among other [3]. HIF-1α protein expression is similar in normoxic and hypoxic conditions but tends to stabilize in hypoxic environments. HIF-1α combines with HIF-1β and activates several HIF-1 target gene to express proteins, such as VEGF [33, 34], which is a significant protein involved in angiogenesis. Overexpression of VEGF can contribute to so many diseases, especially solid-tumors. Tumors are unable to grow beyond a limited size in the absence of adequate blood supply. HIF-1-mediated VEGF leads to angiogenesis, resulting in remittance of hypoxic conditions and tumor metastasis. A rapid rate of tumor growth makes the blood supply again become inadequate, the new circle will occur and finally a balance will be achieved. In Fig. 8, hypoxia-influencing factors are illustrated in a chain. Factors marked with “+” indicate a positive correlation, and factors marked with “-” indicate a negative correlation. The hypoxic level kept spiraling, which was confirmed by BTP phosphorescence in our research that was shown in Fig. 4B.

When tumors are treated by chemotherapy, the hypoxic condition is changed. Cisplatin, a well-studied and commonly used anti-cancer chemotherapy drugs against different human tumors, was used in the present work as a model drug. These series of non-target drugs react *in vivo*, binding to and causing crosslinks of DNA, which ultimately trigger apoptosis. However, previous reports have shown that various cytotoxic chemotherapeutic agents exert anti-angiogenesis effects because the response of chemotherapeutic drugs to vascular endothelial cells were 10–10,000 times more sensitive than that of tumor cells [10, 11]. Although cisplatin is not a target drug of angiogenesis, low-dose metronomic treatment with cisplatin yields anti-angiogenesis effects and restrains the expression of HIF-1α and VEGF [12, 13]. If these proteins are restrained, the angiogenesis is suppressed, but the hypoxic condition may be intensified. In our studies, low-dose metronomic treatment with cisplatin caused anti-angiogenetic effects, and less O_2_ could be delivered to tumors. Finally, BTP phosphorescence intensity increased but the level of HIF-1α was influenced by both chemotherapy and *p*O_2_ in different directions, and its expression would be relied on whose power was stronger.

Angiogenesisis one of the fastest growing fields in biomedical research, and advances in our basic understanding of the process and its clinical application have been rapidly increased. However, the effects of angiogenesis remain controversial. Angiogenesis is a complex, highly orchestrated process that plays a critical role in the normal development and pathophysiology of common diseases. The conventional rationale of anti-angiogenesis therapy is that suppression of angiogenesis and blood supply, which can cause tumor death through ‘‘starvation” [4]. However, the concept of ‘‘vascular normalization,” which was proposed by Jain *et al*. [8], provides another explanation for this phenomenon. Jain *et al*. hypothesized that anti-angiogenesis actually ‘‘normalizes” the tumor vasculature, transiently increases blood perfusion within the tumor, and alleviates hypoxia, thereby enhancing the delivery of oxygen and drugs to tumor cells to increase the malignancies’ response to chemotherapy [35]. These phenotypic changes in vasculature represent a response to changes in the balance of pro- and anti-angiogenesis factors in the tumor tissue [8].

In this current work, we made a proposal that three cases (Type I, II and III) were in the balance of pro- and anti-angiogenesis factors (Table 1). Type I: investigations of predecessors showed that tumor cells become anoxic, inducing cell death [36]. In this case, the anti-angiogenesis affects blood vessels inadequately, causing an increase in the phosphorescence intensity with shortening tumor volumes. However, these anti-angiogenesis drugs are seldom available. Type II: if the anti-angiogenesis effects are not enough, the cells under non-fatal hypoxia may increase the chemotherapy or radiotherapy resistance [37–39], and natural selections will choose the cells with higher tolerance of hypoxia [40]. In this situation, the volume of tumors is inhibited with a low growth speed but the phosphorescence intensity is increased. Type III: vascular normalization has occurred, enhancing the delivery of oxygen and drugs to tumor cells [8], and the phosphorescence intensity is down, but the tumors rapidly grow. Besides, we were unable to find that the volume of tumors decreased with low phosphorescence intensity. We predict it can be observed when tumors are cured.

**Table 1:** Three types in the balance of pro- and anti-angiogenesis.

	Volume of tumors ^a^	Phosphorescence intensity	*p*O_2_ **[41–42]**
Type I	− or +	Strong	<10 mmHg
Type II	+ or++	Medium	10–40 mmHg
Type III	+++	Low	40–60 mmHg
Normal tissues [26]	None	Low	40–60 mmHg

^a^ “^−^” presents a decrease in tumor size and “+” presents an increase in tumor sizes; more “+” marks indicate rapid growth.

According to the data of phosphorescence intensity, *p*O_2_ in tumors could be calculated using the Stern-Volmer equation:

$$\frac{Q_{0}}{Q}=\frac{{\text{τ}}_{\text{0}}}{\text{τ}}=1+K[p{\text{O}}_{2}]=1+\kappa_{q}{\text{τ}}_{0}[p{\text{O}}_{2}]$$ (1)

where *Q*_0_, *κ*_*q*_, and τ_0_ in tumor tissues were obtained from our current and previous work (*Q*_0_ = 7.611×10^9^ phot·cm^-2^·s^-1^, *κ*_*q*_ = 1.2×10^4^ mmHg·s^-1^, τ _0_ = 9.05 μs) [26, 27]. The results are shown in S1 Table. The measurable range is shown in **Fig.** 2B, in which 0–60 mmHg is suitable to be measured with BTP; the relationship between three types in the balance of pro- and anti-angiogenesis is summarized in Table 1. The distinguishing standard of three types (*p*O_2_ in tumors) is set by our data, oxygen dissociation curve of capillary vessels [41], and reference [42].

Other groups showed different results with different dose of treatment, which were shown in S1 Fig. This is the reason that the anti-VEGF agents were singly used, whose therapy effects might be weak and disappointing. Targeting of hypoxia in cancer therapy must consider the combination styles and doses of drugs. For example, chemotherapeutic drugs combined with anti-VEGF inhibitors may cause serious hypoxic conditions in tumors and increase the expression of HIF-1, which induces genes involved in chemotherapy resistance [37–39]. Normalization of the tumor vasculature may also occur since the tumor volume depends on the cytotoxicity of chemotherapeutic drugs. In our study, BTP phosphorescence indicated that low-dose metronomic therapy with cisplatin could adequately suppress angiogenesis, though we did not combine it with VEGF targeting drugs, which belonged to Type II drug effects. However, some cases in our investigation showed that different doses or combinations might cause Type III phenotypic in S1 Fig., and Type I is seldom found because hypoxia-induced cell death is difficult to achieve by chemotherapy or anti-VEGF therapy. For example, the new cisplatin group was close to Type I in the beginning, but became Type II accompanied with therapy, as well as combination group, and the single anti-VEGF group might be in Type III. The phosphorescent data obtained in this work provided quantitative information on processes associated with cancer therapy. In summary, our research proposes a new phosphorescence imaging technique for monitoring hypoxic microenvironment in tumors subjected to chemotherapy. The results of our work can help evaluate therapeutic effects and present new insights into cancer prognosis and treatment.

### Conclusions

A novel method has been developed for monitoring chemotherapy-related changes in tumor hypoxic microenvironment using the hypoxia-sensing probe iridium (III) coordination compound BTP. This probe provides a useful method for quantitative evaluation of changes in tumor hypoxia during chemotherapy. The proposed method is a promising phosphorescence imaging technique for evaluating anti-angiogenetic chemotherapeutic effects *in vivo*.

## Supporting Information

S1 Fig The phosphorescence intensity of tumor imaging of CT-26 xenografts after transplantation into BALB/c nude mice treated with saline and cisplatin with different dose or combined drug treatment (n = 6). Saline (100μL), cisplatin (3 mg·kg^−1^, 100 μL), anti-VEGF (5 mg·kg^−1^, 100μL) or combination (cisplatin 3 mg·kg^−1^+anti-VEGF 5 mg·kg^−1^, 100 μL).
(TIF)

S1 Table The calculated pO2 in tumors through the Stern-Volmer equation in Figs. 4B and S1. (DOCX)
