# Correction: Phosphorescence Monitoring of Hypoxic Microenvironment in Solid-Tumors to Evaluate Chemotherapeutic Effects Using the Hypoxia-Sensitive Iridium (III) Coordination Compound

**DOI:** 10.1371/journal.pone.0126302

**Published:** 2015-04-13

**Authors:** The PLOS ONE Staff

There are errors in the Funding section. The correct funding information is as follows:

"Funding was provided by the National Natural Science Foundation of China, No. 81471771, 81271686, 81000628, 31000379. The funders had no role in study design, data collection and analysis, decision to publish, or preparation of the manuscript.”
