# Supplementary figures and images for: Phosphorescence Monitoring of Hypoxic Microenvironment in Solid-Tumors to Evaluate Chemotherapeutic Effects Using the Hypoxia-Sensitive Iridium (III) Coordination Compound
Source: PLoS One. 2015 Mar 18;10(3):e0121293. doi: 10.1371/journal.pone.0121293 (PMC4365010; doi:10.1371/journal.pone.0121293)

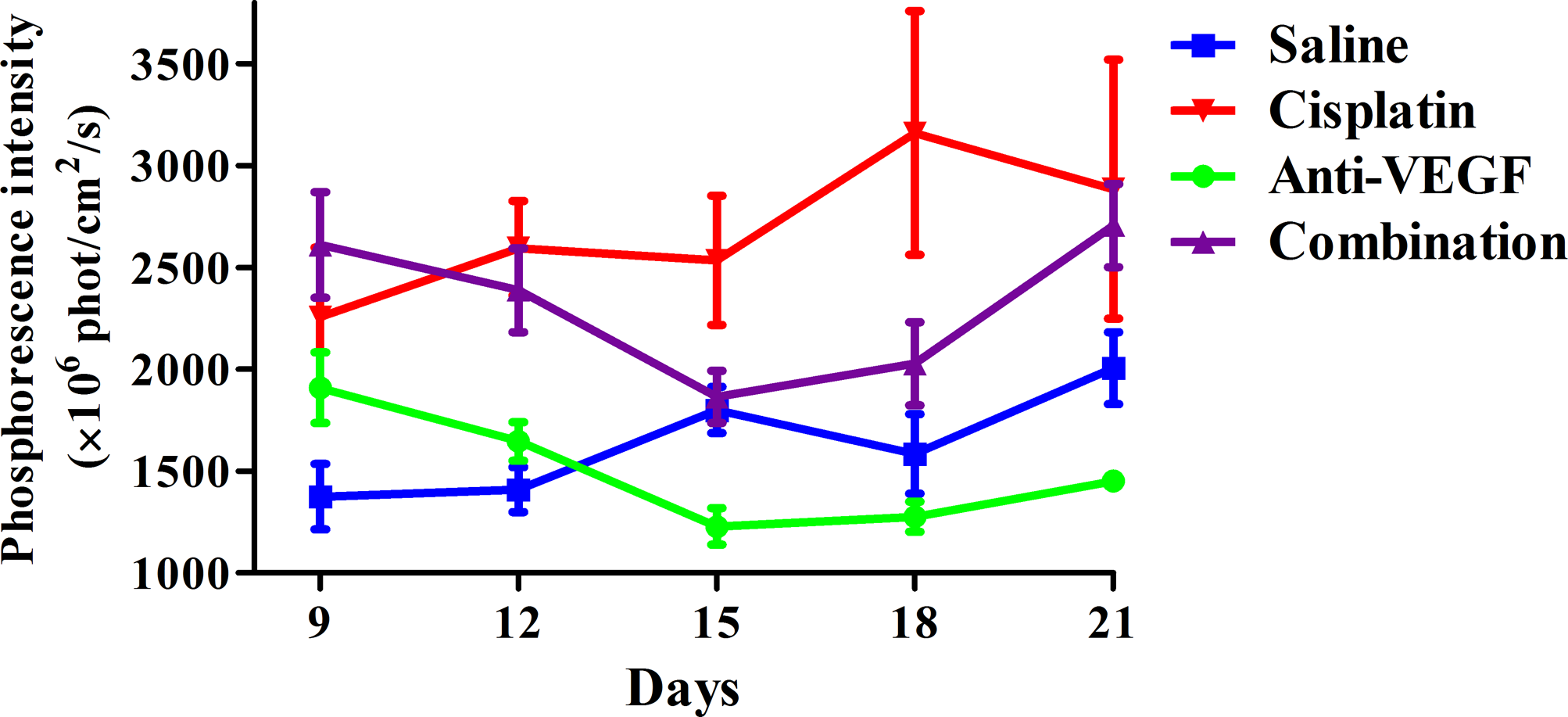

Supplement: S1 Fig — Saline (100μL), cisplatin (3 mg·kg−1, 100 μL), anti-VEGF (5 mg·kg−1, 100μL) or combination (cisplatin 3 mg·kg−1+anti-VEGF 5 mg·kg−1, 100 μL). (TIF) [file pone.0121293.s001.tif]
