# Supplementary material for: Phosphorescence Monitoring of Hypoxic Microenvironment in Solid-Tumors to Evaluate Chemotherapeutic Effects Using the Hypoxia-Sensitive Iridium (III) Coordination Compound
Source: PLoS One. 2015 Mar 18;10(3):e0121293. doi: 10.1371/journal.pone.0121293 (PMC4365010; doi:10.1371/journal.pone.0121293)
Supplement: S1 Table — (DOCX) [file pone.0121293.s002.docx]

**Table S1.** The calculated *p*O2 in tumors through the Stern-Volmer equation in Figures 4b and S1

| **Days**  **Groups** | | ***p*O_2_ (mmHg)** | | | | |
| --- | --- | --- | --- | --- | --- | --- |
|  |  | 9 | 12 | 15 | 18 | 21 |
| Figure 4b | Saline | 60.38 | 51.50 | 51.91 | 42.20 | 42.77 |
|  | Cisplatin | 64.64 | 48.57 | 50.67 | 31.23 | 27.44 |
| Figure S1 | Saline | 41.76 | 40.52 | 29.73 | 34.99 | 25.73 |
|  | Cisplatin | 21.85 | 17.80 | 18.44 | 12.96 | 15.09 |
|  | Anti-VEGF | 27.49 | 33.36 | 47.84 | 45.69 | 39.07 |
|  | Combination | 17.62 | 20.13 | 28.38 | 25.36 | 16.69 |
